# The Effect of Buffers on Weak Acid Uptake by Vesicles

**DOI:** 10.3390/biom9020063

**Published:** 2019-02-13

**Authors:** Christof Hannesschlaeger, Thomas Barta, Hana Pechova, Peter Pohl

**Affiliations:** Institute of Biophysics, Johannes Kepler University Linz, Gruberstr. 40, 4020 Linz, Austria; Christof.Hannesschlaeger@jku.at (C.H.); Thomas.Barta@jku.at (T.B.); hanpechova@gmail.com (H.P.)

**Keywords:** weak acid permeation, passive membrane permeability, membrane permeation, stopped flow, buffer, vesicles

## Abstract

The assessment of weak acid membrane permeability (*P*_m_) frequently involves large unilamellar vesicles. It relies on measurements of the intravesicular pH drop, ΔpH_in_, in response to a sudden augmentation of external acid concentration. However, ΔpH_in_ may be primarily governed by non-instantaneous protonation and deprotonation reactions of (i) the acid itself, (ii) the buffer molecules, and (iii) the fluorescent pH reporter dye. Moreover, buffer concentration and acid gradient also serve as determinants of ΔpH_in_, as we show here. The uniexponential time constant (τ) of ΔpH_in_(t) is an invalid measure of *P*_m_ as Arrhenius plots of *P*_m_ and τ reveal different activation energies for acid influx. We calculate *P*_m_ by fitting a mathematical model to experimental stopped-flow traces. The model takes into account not only the time course of total internal buffer capacity but also (i) water self-dissociation, (ii) volume changes due to acid induced osmotic water flow, and (iii) the spontaneous membrane proton leak. It allows extracting a *P*_m_ of 30.8 ± 3.5 μm/s for formic acid for 1,2-dioleoyl-*sn*-glycero-3-phosphocholine (DOPC) vesicles.

## 1. Introduction

Many pharmacologically important substances belong to the class of weak acids and bases [1,2]. Such drugs are appealing because they may permeate membranes in their neutral form and yet attain high water solubility in their charged form at physiological pH values [3]. Cellular uptake of these substances is intricately linked to protonation and deprotonation reactions [4]. Upon permeating membranes, weak acids augment the pH of the solution they leave and acidify the solution they enter:

(1) $$\left[AH\right]\begin{array}{c} k^{+}\\ ⇌\\ k^{-} \end{array}\left[A^{-}\right]+\left[H^{+}\right],$$

where $k^{+}$ and $k^{-}$ denote the reaction rates for the deprotonation and protonation reaction, respectively.

These pH changes are used to assess weak acid membrane permeability, $P_{m}$ [5]. In the most widely used assay, large unilamellar lipid vesicles (LUV) are exposed to a jump in external [AH] concentration and $P_{m}$ is derived from the time constant (τ) of exponential intravesicular pH change [6,7]. However, the assumed proportionality between $P_{m}$ and τ is only valid in a very limited set of experimental conditions as demonstrated by the following derivation:

The flux density (J) of a weak acid across the membrane of area (S) into a spherical vesicle of radius (r) is defined as:

(2) $$J=-S\cdot P_{m}\cdot \Delta \left[AH\right],$$

where $\Delta \left[AH\right]={\left[AH\right]}_{in}-{\left[AH\right]}_{out}$ is the transmembrane gradient of the protonated acid. A positive value for J corresponds to influx. Per definition, J must also be equal to the first-time derivative of the amount (*n*) of molecules (in moles) that cross the membrane per unit area. Exploiting ${\left[AH\right]}_{in}=n_{AH,in}/V$, Equation (2) may be transformed into:

(3) $$\frac{d{\left[AH\right]}_{in}}{dt}=-\frac{S}{V}\cdot P_{m}\cdot \left({\left[AH\right]}_{in}-{\left[AH\right]}_{out}\right).$$

Solving Equation (3) with respect to the boundary conditions that (i) ${\left[AH\right]}_{in}=0$ for *t* = 0, (ii) ${\left[AH\right]}_{out}=\mathrm{constant}$, i.e., time invariant and (iii) vesicle volume is independent on time (i.e., S/V=3/r at all times), i.e., there is no acid flux induced osmotic water flow, yields:

(4) $${\left[AH\right]}_{in}={\left[AH\right]}_{out}\cdot \left(1-e^{-\frac{3\cdot P_{m}}{r}\cdot t}\right).$$

Equation (4) is valid for substances that do not undergo chemical reactions. Weak acids with a pK value below the intravesicular pH value ($pH_{in}$) suffer a net proton release upon arrival. Accounting for the fraction of deprotonated acid molecules transforms Equation (4) into:

(5) $$\frac{d{\left[AH\right]}_{in}}{dt}=-\frac{S}{V}\cdot P_{m}\cdot \left({\left[AH\right]}_{in}-{\left[AH\right]}_{out}\right)\cdot \frac{1}{1+10^{pH_{in}-pK}}$$

Equation (5) is only valid if the chemical reactions are not rate limiting, i.e., if proton uptake and release reactions are in equilibrium. Equation (5) can easily be solved assuming a time invariant (constant) $pH_{in}$:

(6) $$\begin{array}{l} {\left[AH\right]}_{in}={\left[AH\right]}_{out}\cdot \left(1-e^{-\frac{3\cdot P_{m}}{r}\cdot \frac{1}{1+10^{pH_{in}-pK}}\cdot t}\right)={\left[AH\right]}_{out}\cdot \left(1-e^{-\frac{t}{\tau }}\right),\;\\ \mathrm{with}\;\;\tau ∶=\frac{r\cdot \left(1+10^{pH_{in}-pK}\right)}{3\cdot P_{m}}, \end{array}$$

where τ is an exponential time constant. Thus, Equation (6) is not valid in experiments where significant changes of intravesicular pH ($pH_{in}$) take place. That is, Equation (6) does not describe experiments which rely on measurements of a significant $pH_{in}$ drop, $\Delta pH_{in}$, for determining $P_{m}$.

**Figure 1:**
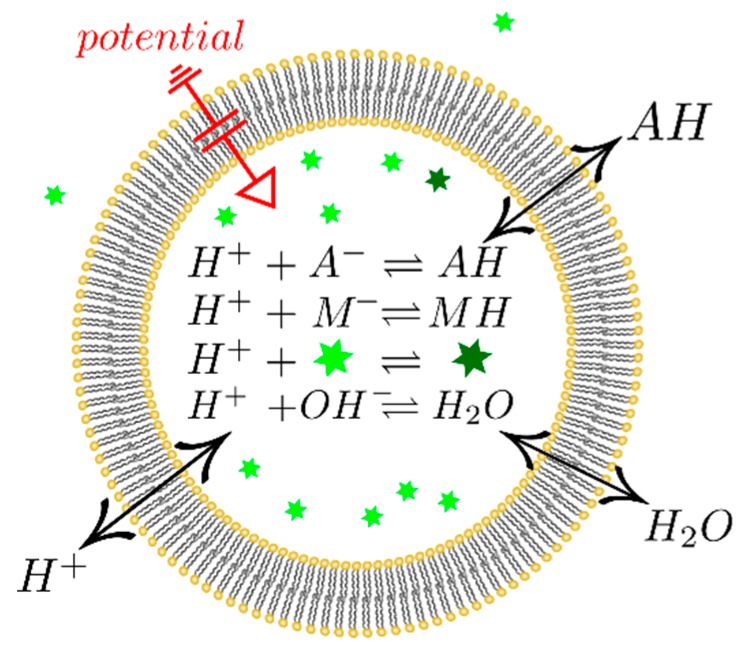
Graphic representation of the experimental system. Weak acid, buffer, water, and fluorophore participate in protonation/deprotonation reactions. Upon protonation, the fluorophore (bright green star) ceases to fluoresce (dark green star). Weak acid, proton, and water fluxes across the membrane are driven by the respective electrochemical gradients. Charge transfer by protons gives rise to a transmembrane potential.

Another problem of Equation (6) is its neglect of accompanying chemical reactions. That is, water hydrolysis as well as proton uptake and release by buffer molecules or by fluorescent dyes (Figure 1) may confound the analysis. In other words, the very method of exploiting protonation and deprotonation reactions of encapsulated pH-sensitive dyes as readout may introduce a systematical error to the assessment of $P_{m}$. Commonly, the dye has a much higher pK value than the weak acid itself, and thus proton release from the dye molecules may be too slow to measure acid membrane translocation. The resulting liability of Equation (6) to misinterpretations has previously been recognized [8]. The same considerations are valid for buffer molecules with higher pK values. In addition, Equation (6) ignores the importance of buffer capacity in vesicular uptake experiments. Its significance has previously been demonstrated for weak acid and weak base permeation across planar bilayers [9,10,11]. This paper aims at adopting our previously published mathematical model [9,10,11] to describe buffer effects on weak acids flux into lipid vesicles.

An alternative approach of measuring $P_{m}$ is based on the assessment of osmotic water flow that accompanies acid efflux from vesicles. Vesicle shrinkage can be assessed by measuring the intensity of scattered light, i.e., it is possible to obtain $P_{m}$ in fluorophore-free experiments [12]. Of course, that assay requires high values of membrane water permeability $\left(P_{f}\right)$ to ensure that the osmotic water flux is not rate limiting. Consequently, reconstitution of water channels (aquaporins) into the vesicular membrane may be necessary. The drawback of that approach is that the acid has to be present in significant amounts to induce observable volume changes. To prevent $pH_{in}$ from increasing during efflux, sizable amounts of encapsulated buffer molecules are required. In consequence, the osmotic assay also requires buffer-reactions to be considered.

Our analysis shows that the neglect of buffer molecules may result in misinterpretations of the experimental results. Most importantly, the commonly assumed proportionality between τ and $P_{m}$ is not guaranteed. That is, changes in buffer capacity may well give rise to changes in τ while $P_{m}$ remains unaltered.

## 2. Materials and Methods

### 2.1. Computation

We developed a mathematical model for weak acid transport across membranes that accounts both for the accompanying water flux and the presence of buffer. The model assumes that only the protonated form of the weak acid is membrane permeable. Since most of the acids are negatively charged at physiological pH values, protonation and deprotonation reactions are of utmost importance. They result in the following time derivatives for the concentration of the acid, its conjugated base and protons:

(7) $$\frac{d\left[AH\right]}{dt}=-\frac{d\left[A^{-}\right]}{dt}=-\frac{d\left[H^{+}\right]}{dt}=-k^{+}\cdot \left[AH\right]+k^{-}\cdot \left[A^{-}\right]\cdot \left[H^{+}\right],$$

(8) $$K=10^{-pK}=\frac{k^{+}}{k^{-}}.$$

The ratio of protonation and deprotonation rates is the acid dissociation constant *K* (in M), which is usually indicated in terms of the acid’s pK value (Equation (8)).

Acid uptake by vesicles results in a difference of osmolarities $\Delta Osm=Osm_{in}-Osm_{out}$. The resulting water flux gives rise to a change of volume *V*. For the sake of simplicity, ΔOsm comprises the sum of the concentrations of osmotically active substances:

(9) $$\frac{dV}{dt}=\dot{V}=S\cdot V_{w}\cdot P_{f}\cdot \Delta Osm,$$

where $V_{w}=0.018\;L/\mathrm{mol}$ is the molar volume of water and $P_{f}$ is the membrane water permeability. Changes of vesicle volume act to alter the concentration of the enclosed substances. In addition, both the expenditure in the course of chemical reactions and the diffusion across the membrane result in concentration changes:

(10) $$\frac{dn_{AH,in}}{dt}=\left(-k_{AH}^{+}\cdot {\left[AH\right]}_{in}+k_{AH}^{-}\cdot {\left[A^{-}\right]}_{in}\cdot {\left[H^{+}\right]}_{in}\right)\cdot V-S\cdot P_{m}\cdot \left({\left[AH\right]}_{in}-{\left[AH\right]}_{out}\right),$$

where $n_{AH,in}$ is the amount of AH in moles. Since the charged $A^{-}$ is treated as membrane impermeable, its time derivative is:

(11) $$\frac{d\left[A^{-}\right]}{dt}=k_{AH}^{+}\cdot \left[AH\right]-k_{AH}^{-}\cdot \left[A^{-}\right]\cdot \left[H^{+}\right].$$

For the non-permeating buffer ($M^{-}$, MH) and the pH-dependent fluorophore (deprotonated and protonated carboxyfluorescein $CF^{-}$, CFH) similar relations hold. In addition, the self-ionization of water may also serve to release protons:

(12) $$K_{w}=\frac{\left[H_{2}O\right]\cdot k_{w}^{+}}{k_{w}^{-}}.$$

Water with a concentration of $\left[H_{2}O\right]=\;V_{w}^{-1}$ dissociates at rate $k_{w}^{+}$. Recombination of protons and hydroxide occurs at rate $k_{w}^{-}$. $K_{w}$ is the water equilibrium constant. Water self-dissociation changes the hydroxide concentration $\left[OH^{-}\right]$ and contributes to the change in $\left[H^{+}\right]$:

(13) $$\frac{d\left[OH^{-}\right]}{dt}=\frac{d\left[H^{+}\right]}{dt}=k_{w}^{+}\cdot \left[H_{2}O\right]-k_{w}^{-}\cdot \left[OH^{-}\right]\cdot \left[H^{+}\right].$$

Protons possess high membrane permeability [13,14]. As we show in the experimental section, membrane translocation of all other charged species can be neglected. Consequently, we find the decrease of the intravesicular amount $\left(n_{cc}\right)$ of protons due to proton egress from the Goldman–Hodgkin–Katz flux equation [15,16,17] as:

(14) $$\frac{dn_{cc}}{dt}=-U\cdot S\cdot \frac{P_{H^{+}}\cdot F}{R\cdot T}\cdot \frac{{\left[H^{+}\right]}_{in}-{\left[H^{+}\right]}_{out}\cdot \exp\left(-\frac{U\cdot F}{R\cdot T}\right)}{1-\exp\left(-\frac{U\cdot F}{R\cdot T}\right)},$$

where F, R, T, U are Faraday’s constant, gas constant, absolute temperature, and the transmembrane voltage, respectively.

In addition to proton egress, the luminal proton concentration is also altered by chemical reactions. Hence the change in luminal proton concentration is not a measure of the charge that loads the capacitor. Only *n*_cc_ gives rise to U by charging the membrane capacitor: c=Q/(S·U), where Q (in coulombs) is the charge imbalance between the two sides of the capacitor. Consequently, we find the following expression for U:

(15) $$U=-\frac{n_{cc}\cdot F}{S\cdot c}.$$

The negative sign in Equation (15) indicates the directionality of the electrical potential. 

Accounting for (i) water self-dissociation (Equation (13)), (ii) proton uptake and release reactions of membrane impermeable buffer molecules ($M^{-}$, MH with deprotonation, protonation rates $k_{M}^{-}$, $k_{M}^{+}$, respectively—as in Equation (7)), and (iii) pH dependent fluorophore ($CF^{-}$, CFH) deprotonation and protonation rates, $k_{CF}^{-}$, $k_{CF}^{+}$, respectively), the time derivative of intravesicular proton amount $n_{H^{+},in}$ adopts the form:

(16) $$\frac{dn_{H^{+},in}}{dt}=V\cdot \left(k_{w}^{+}\cdot \left[H_{2}O\right]-k_{w}^{-}\cdot {\left[OH^{-}\right]}_{in}\cdot {\left[H^{+}\right]}_{in}+k_{AH}^{+}\cdot {\left[AH\right]}_{in}-k_{AH}^{-}{\left[A^{-}\right]}_{in}\cdot {\left[H^{+}\right]}_{in}\;+\;k_{CF}^{+}\cdot {\left[CFH\right]}_{in}-k_{AH}^{-}\cdot {\left[CF^{-}\right]}_{in}\cdot {\left[H^{+}\right]}_{in}+k_{M}^{+}\cdot {\left[MH\right]}_{in}-k_{M}^{-}{\left[M^{-}\right]}_{in}\cdot {\left[H^{+}\right]}_{in}\right)+\frac{dn_{cc}}{dt}.$$

The initial conditions for solving the system of differential Equations (10)–(16) are given by the initial volume of the vesicles and the concentrations of salt, buffer, and weak acid. Since the pH of the solution, in which the vesicles are formed, is adjusted with HCl, the actual amount of chloride inside the vesicles is calculated assuming electrical neutrality. All values used for the computation are summarized in Table 1. The acid deprotonation rates are set to $k^{+}=10^{10.5-pK}\;s^{-1}$ [18] if not otherwise indicated. All protonation rates are presumed to be diffusion limited, i.e., $k^{-}\approx 2\times 10^{10}{\;s}^{-1}M^{-1}$ [18].

The measured fluorescence intensity (*I*) depends on $\left[CF^{-}\right]\left(t\right)$, since carboxyfluorescein is only fluorescent when deprotonated [19]. Variations in $\left[CF^{-}\right]\left(t=0\right)$ between different runs of the experiment are accounted for by defining the normalized experimental fluorescence intensity ($I_{exp}$) as the ratio of *I* values at time *t* and time *t* = 0: $I_{exp}\left(t\right)=I\left(t\right)/I\left(t=0\right)$. The *I* value at t=0 is not attainable by experimental observation because mixing of vesicle suspension with acid solution is not instantaneous, i.e., time $t_{mixing}$ elapses before mixing is completed. Consequently, I(t=0) is obtained by first fitting a monoexponential function to *I*(*t*) for $t_{mixing}$ ≤ *t* ≤ 0.1 s and second extrapolating this fit to *I*(*t* = 0).

Our model predicts theoretical fluorescence intensity ($I_{CF^{-}\left(t\right)}$) by assuming proportionality to calculated $\left[CF^{-}\right]\left(t\right)$ values (Equations (10)–(16)). Comparing prediction and experiment requires normalization of $I_{CF^{-}\left(t\right)}$. The procedure must accommodate the absolute value $I_{a}$ of time invariant background intensity. This value ($I_{a})$ originates from detector dark counts, unspecific fluorescence, and non-encapsulated carboxyfluorescein:

(17) $$I_{theor}\left(t\right)=\frac{I_{CF^{-}\left(t\right)}+I_{a}}{I_{CF^{-}\left(t=0\right)}+I_{a}}=\frac{a\left[CF^{-}\right]\left(t\right)+I_{a}}{a\left[CF^{-}\right]\left(t=0\right)+I_{a}},$$

where $I_{theor}\left(t\right)$ is the normalized theoretical fluorescence intensity. Substituting proportionality factor *a* for the ratio *I*_max_/[CF]_tot_ of maximum fluorescence intensity and total concentration of encapsulated dye yields:

(18) $$I_{theor}\left(t\right)=\frac{\left[CF^{-}\right]\left(t\right)/{\left[CF\right]}_{tot}+I_{b}}{\left[CF^{-}\right]\left(t=0\right)/{\left[CF\right]}_{tot}+I_{b}},$$

where $I_{b}=I_{a}/I_{max}$ is the relative background fluorescence intensity.

The following three steps serve to extract $P_{m}$ from $I_{exp}\left(t\right)$:

1. Numerical calculation of $\left[CF^{-}\right]\left(t,P_{m}\right)$, the time course of the concentration of fluorescent dye as function of $P_{m}$ for given initial conditions.
2. $I_{theor}\left(t,P_{m},I_{b}\right)$ is calculated from $\left[CF^{-}\right]\left(t,P_{m}\right)$ for varying $I_{b}$.
3. $I_{theor}\left(t,P_{m},I_{b}\right)$ is fitted to $I_{exp}\left(t\right)$ using $P_{m}$ and $I_{b}$ as fitting parameters.

Step 1 is accomplished in Wolfram Mathematica 11.2 [20]. With the “ParametricNDSolveValue” routine, a parametric solution for $\left[CF^{-}\right]\left(t,P_{m}\right)$ is obtained with respect to the initial conditions employing the built-in implicit differential-algebraic (IDA) solver. This solver is a part of the SUite of Nonlinear and DIfferential/ALgebraic Equation Solvers (SUNDIALS, [21]).

In step 2, $\left[CF^{-}\right]\left(t,P_{m}\right)$ is inserted into Equation (18) for $I_{theor}\left(t\right)$, which is evaluated in a table for different $P_{m}$ and $I_{b}$. Employing Mathematica’s “Interpolation” routine results in an interpolating formula for $I_{theor}\left(t,P_{m},I_{b}\right)$.

In step 3, a global fit over $I_{exp}\left(t\right)$ traces for different initial conditions is performed to find robust *P*_m_ values. For that, $I_{theor}\left(t,P_{m},I_{b}\right)$ for the respective initial conditions are used in a “NonlinearModelFit” routine.

#### 2.1.1. Temperature Correction

Equilibrium and kinetic parameters change with temperature. Calculating the deprotonation rate at the temperature of interest from the deprotonation rate $k^{+0}$ at a known reference temperature $T^{0}$ (22 °C) requires to take into account the activation energy of proton diffusion $E_{H^{+}}=4.3\;k_{B}T$ [22]:

(19) $$k^{+}\left(T\right)=10^{-pK\left(T\right)}\cdot k^{-}\left(T\right)=10^{-pK^{0}}\cdot 10^{-dpK\cdot \left(T-T^{0}\right)}\cdot k^{-0}\cdot e^{-\frac{E_{H^{+}}}{k_{B}T}\cdot \left(\frac{1}{T}-\frac{1}{T^{0}}\right)}\;=k^{+0}\cdot e^{-\frac{E_{H^{+}}}{k_{B}T}\cdot \left(\frac{1}{T}-\frac{1}{T^{0}}\right)}\cdot 10^{-dpK\cdot \left(T-T^{0}\right)},$$

where dpK is shift of the pK value at temperature T. $V_{w}$ is treated as temperature independent as it varies by less than one percent [23] in the temperature range of interest. $P_{f}$ is corrected by using Arrhenius’ equation and a typical activation energy of 12 kcal/mol for natural lipid mixtures (*Escherichia coli* polar lipid extract) and synthetic phospholipids (1,2-diphytanoyl-*sn*-glycero-3-phosphocholine) [24].

#### 2.1.2. Additional Assumptions in the Model

In addition to those already mentioned, the model is based on the following assumptions:

- The time it takes a molecule to pass a membrane of thickness (L) can be neglected. The time $T_{Lag}$ between the onset of the Fickian flux (Equation (2)) upon the application of $\Delta \left[AH\right]=\left[AH\right]-{\left[AH\right]}_{out}$ is estimated as [25]:
  (20) $$T_{Lag}=\frac{L^{2}}{6\cdot D_{m}}\approx \frac{10\cdot L^{2}}{6\cdot D_{aq}},$$
  $D_{m}$ denotes the diffusion coefficient of a substance within the membrane. It can be approximated to be equal to ~1/10 of the aqueous diffusion coefficient $D_{aq}$ [26]. $T_{Lag}$ amounts to ~40 ns for *L* = 5 nm and $D_{aq}$ = $10^{-5}{\mathrm{cm}}^{2}/s$. Thus, $T_{Lag}$ ≪ $t_{mixing}$ indicating that the application of Δ[AH] gives rise to an instantaneous *J*.
- Solute bulk concentrations remain unaltered throughout the experiment because only 1/1000 of the volume of the suspension is encapsulated by vesicles. This can be estimated from (i) the mass concentration of lipid in the measurement cuvette (about 0.3 mg/mL), (ii) the molar mass of 1,2-dioleoyl-*sn*-glycero-3-phosphocholine (DOPC, 786 g/mol), and (iii) an area per lipid of approximately $70\;{Å}^{2}$ [27].
- Diffusion through stagnant water layers (unstirred layers) in the immediate membrane vicinity can be neglected because their width does not exceed the vesicle diameter [28]. A molecule with $D_{aq}$ = $10^{-5}{\mathrm{cm}}^{2}/s$ crosses this distance within a few µs. $t_{mixing}$ is orders of magnitude larger.
- Carboxyfluorescein (CF) residues that display acidic pK’s are neglected since they do not contribute to buffer capacity at experimental pH. Only $pK_{CF}$ ~6.45 is considered. It is well described by the Henderson–Hasselbalch equation (Figure S1).
- For the same reason, the highly acidic pK of DOPC (2.25; [18]) is neglected.

#### 2.1.3. Activation Energy of Membrane Permeation

The energy barrier that a membrane imposes to permeation is experimentally accessible via temperature dependent measurements of $P_{m}$ [37,38] or a parameter that is proportional to $P_{m}$. Commonly, the activation energy $\left(E_{A}\right)$ is extracted via an Arrhenius plot (Equation (21); [39]):

(21) $$P_{m}=A\cdot \exp\left(-\frac{E_{A}}{R\cdot T}\right),$$

where A is some temperature independent constant.

If the inverse value of time constant τ (Equation (6)) was proportional to $P_{m}$ (Equation (6)), $E_{A}$ derived from an Arrhenius plot for $\tau^{-1}$ must be the same as derived from $P_{m}$.

### 2.2. Buffers

All buffers are prepared with MilliQ water (Millipore; Billerica, MA, USA) with a specific resistance of 18 MΩ cm. Chemicals are purchased from Sigma-Aldrich (St. Louis, MO, USA). The intravesicular and extravesicular buffers contain 100 mM KCl, and 5, 10, or 20 mM MES (2-(*N*-morpholino)ethanesulfonic acid). They are adjusted to pH 7 by HCl addition. The intravesicular solution additionally contains 0.5 mM 5(6)-carboxyfluorescein (CF).

### 2.3. Large Unilamellar Vesicles 

A mass of 5 mg of DOPC (Avanti Polar Lipids, Alabaster, AL, USA) are dissolved in chloroform and added to a glass test tube. Evaporation on a rotavapor for at least 45 min at about 20 mbar vacuum results in a thin lipid film on the glass wall. Rehydration of the lipid film to a 10 mg/mL suspension allows obtaining LUVs by extrusion through 100 nm wide pores. External CF is removed by a Sephadex desalting column (PD-10, GE Healthcare, Chicago, IL, USA). Fresh vesicles are prepared daily.

### 2.4. Stopped Flow Experiments

A 50 mM stock solution of sodium formate is prepared in the external buffer and mixed with an eight-fold diluted vesicle suspension by a stopped-flow device (SFM-300, bio-logic, Seyssinet-Pariset, France). A 75 mW xenon lamp excites the sample at 480 nm at 4 nm bandwidth. A photomultiplier tube collects the light that is emitted at a right angle behind a 515 nm long-pass filter. Fluorescence intensity is hardware filtered with 300 µs and is sampled at a rate of 1 ms. Each curve is recorded at least six times and then averaged. Per trace, 151 µL total volume are pushed through the cuvette at a flow rate of 9 mL/s resulting in a dead time ($t_{mixing}$) of 1.7 ms.

### 2.5. Dynamic Light Scattering 

A DelsaNano HC particle analyzer (Beckman Coulter; Brea, CA, USA) measures the intensity of scattered light to extract the liposome size. Vesicles radius is derived from the mean of the volume distribution, since the amount of encapsulated dye scales with vesicle volume.

### 2.6. Estimation of Proton Permeability

Membranes are orders of magnitude more permeable to protons than to other cations [13] suggesting that a proton leak might occur. The latter is worth estimating since *I* depends on proton concentration: We subject the vesicular membrane to a pH gradient by pipetting HCl to a continuously stirred solution and the fluorescence spectrophotometer (HITACHI F2700; Tokyo, Japan) tracks the decay of *I*. Grid monochromators are set to 480 and 520 nm for excitation and emission, respectively. The bandwidths are restricted to 5 nm. We added the potassium ionophore valinomycin to a final aqueous concentration of 1µM. It clamped the membrane potential to a value that was given by the transmembrane concentration gradient of potassium [40], i.e., to a value that was not significantly different from zero. This conclusion is based on the Goldman–Hodgkin–Katz equation that calculates the membrane potential from the permeabilities of the different ionic species and their concentrations. Since the potassium concentration in our experiments exceeded the proton concentration by roughly six orders of magnitude, the valinomycin induced enhancement of proton permeability by a factor of two [13] remains without effect on the membrane potential.

## 3. Results

### 3.1. Vesicle Size

**Figure 2:**
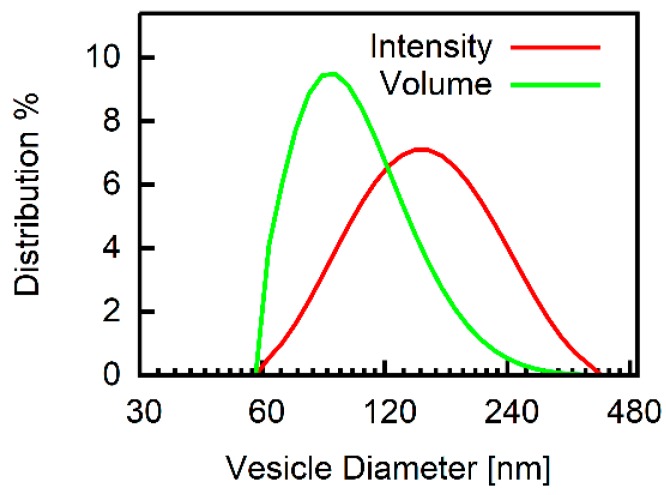
Vesicle size. Representative semi-logarithmic intensity and volume distributions (107.4 ± 38.4 nm) from Dynamic Light Scattering (DLS) measurements. 1,2-dioleoyl-*sn*-glycero-3-phosphocholine (DOPC) vesicles (100 mM KCl, 0.5 mM carboxyfluorescein (CF), 5 mM MES pH 7) after extrusion through a 100 nm filter and removal of external CF.

The volume distribution returned by the Dynamic Light Scattering (DLS) measurements indicated a mean diameter of about 110 nm for DOPC vesicles. For each preparation, the diameter was determined and used for the calculations (e.g., Equation (10)). A representative measurement is shown in Figure 2.

### 3.2. Estimate for Proton Permeability

**Figure 3:**
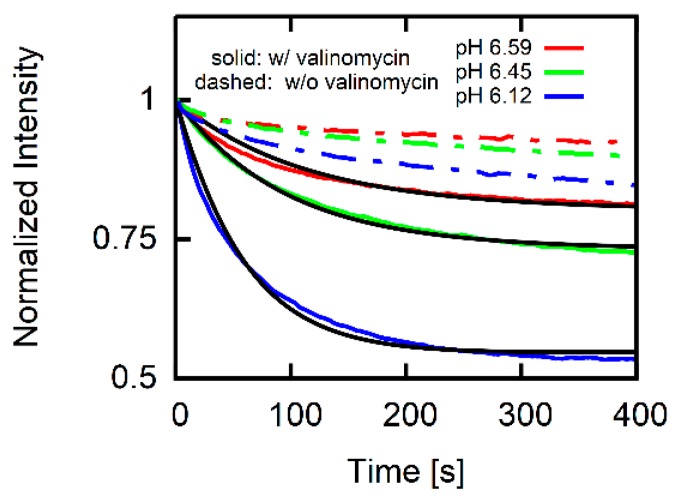
Assessment of membrane proton permeability. DOPC vesicles in 100 mM KCl, 2 mM MES, pH 7 (inside additionally 0.5 mM CF) are exposed to decreased external pH (denoted in the key) by addition of HCl. The potassium ionophore valinomycin (final concentration 1 µM) clamps the transmembrane potential to almost zero (solid colored lines). A global fit of $I_{theor}$ (black lines) to $I_{exp}$ (colored lines) reveals a proton permeability of $P_{H^{+}}=3.5\cdot 10^{-5}\;\mathrm{cm}/s$ in the presence (w/) of valinomycin. The kinetics are slower in the absence (w/o) of valinomycin (dashed lines) confirming that membrane potential U > 0 mV hinders proton translocation across the membrane.

**Figure 4:**
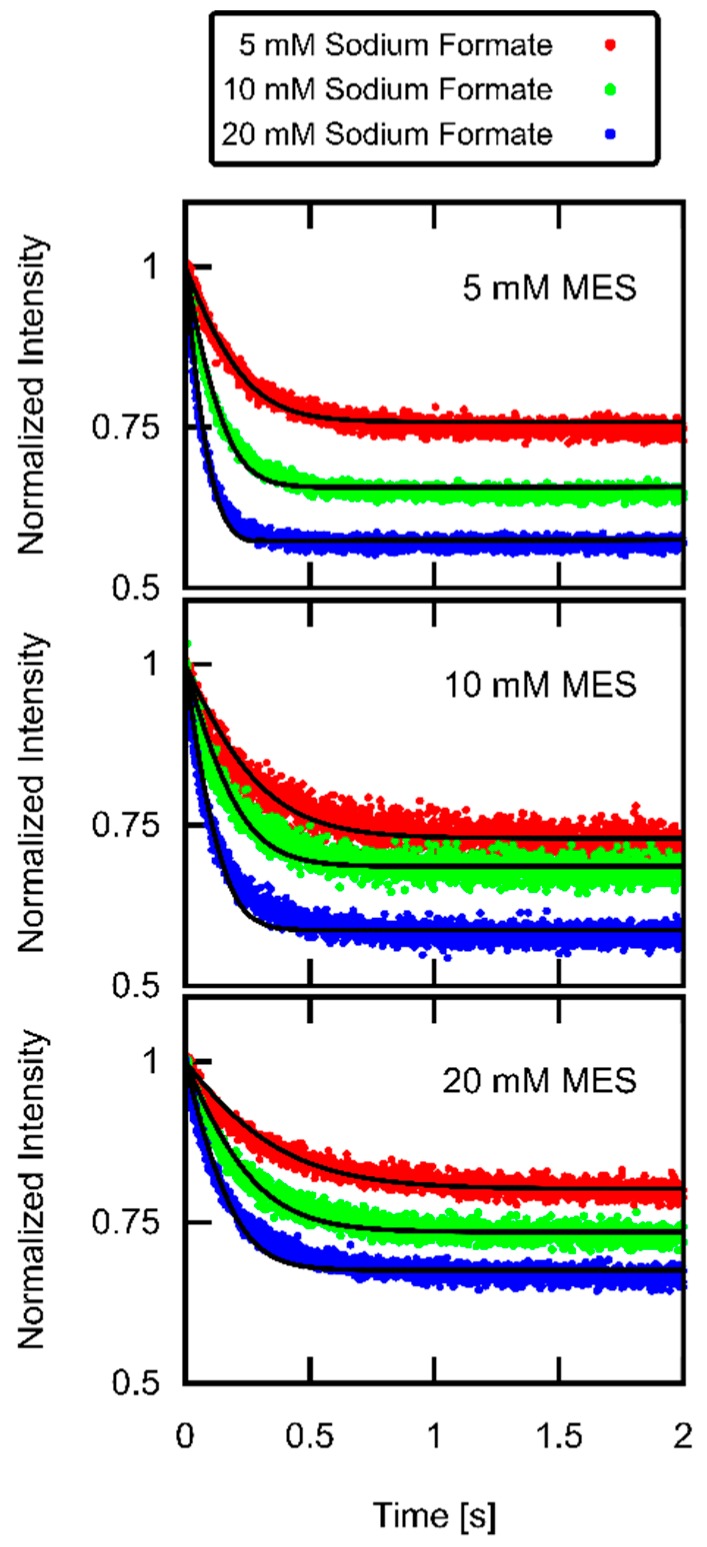
Weak acid uptake. DOPC vesicles loaded with 100 mM KCl and 0.5 mM CF (pH 7) were objected to gradients of sodium formate in 100 mM KCl (pH7) at time 0. Initial intravesicular and extravesicular MES concentrations amounted to the values indicated in the panels. Global (per panel) fits of $I_{theor}$ (black lines) to $I_{exp}$ (colored lines) resulted in formic acid permeabilities $P_{m}$ of 29.1, 34.9, and 33.3 µm/s for the upper, middle, and lower panels, respectively.

Acidification of the external solution leads to a decline in $I_{exp}$ (Figure 3). We apply the above developed system of differential Equations (10)–(16) assuming that 1 μM of the potassium ionophore valinomycin clamps U to almost zero. Accounting for the absence of AH (${\left[AH\right]}_{out}=\left[AH\right]\left(t=0\right)=0$) and substituting Equation (14) for a Fickian flux equation for protons yields $P_{H^{+}}=3.5\cdot 10^{-5}\;\mathrm{cm}/s$. The value is in the range of previously reported values [41]. Thus, $P_{H^{+}}$ is negligibly small since AH uptake leads to 100 times faster acidification of the vesicular interior (Figure 4). Performing the same experiment without valinomycin (dashed lines in Figure 3) yielded a much slower decaying pH since U < 0 mV. This observation supports our assumption that the permeation of counterions (e.g., chloride) does not significantly contribute to U albeit counterions are present in much larger concentrations than protons.

### 3.3. Formic Acid Membrane Permeability of DOPC

Objecting CF loaded vesicles to a jump of external formic acid concentration leads to a drop in $I_{exp}$ (Figure 4). For each buffer concentration, we obtained $P_{m}$ by globally fitting $I_{theor}$ (Equation (18)) to $I_{exp}$ (single panels in Figure 4). The average (*n* = 4) of all $P_{m}$ values at different buffer concentrations results in $P_{m}=30.8\pm 3.5\;µm/s$.

**Figure 5:**
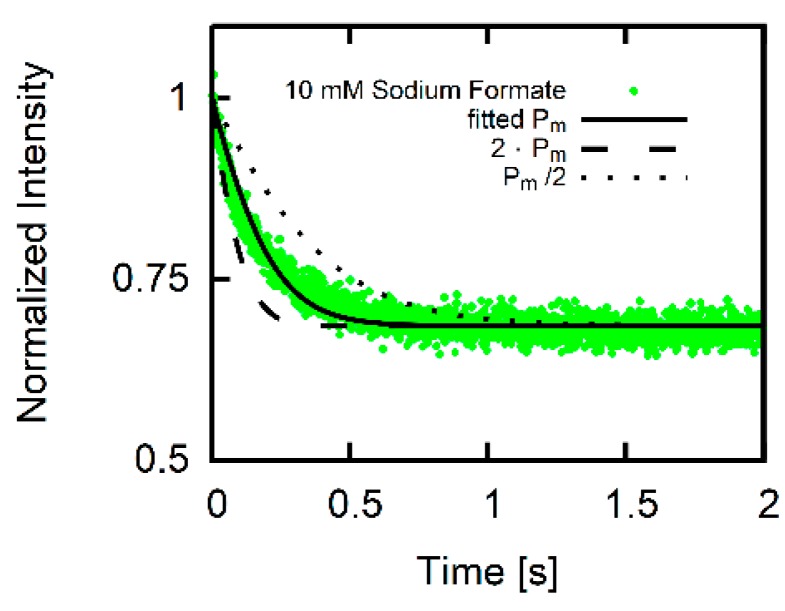
Sensitivity of the fit to $P_{m}.$ Fluorescence trace for 10 mM sodium formate and 10 mM MES (middle trace of middle panel in Figure 4) together with the result of the fitting procedure (solid black line). Curves for double (dashed black line) or half (dotted black line) the fitted $P_{m}$ reveals that the algorithm calculates traces sensitive for changes in $P_{m}$.

In order for our $P_{m}$ value to be correct, AH membrane translocation must be rate limiting. In other words, proton uptake and release must occur at higher rates [8]. Model calculations confirm that the time constant of $I_{theor}$’s decay is sensitive to $P_{m}$. That is, doubling $P_{m}$ or cutting it in half results in an obvious misfit of $I_{theor}$ to $I_{exp}$ (Figure 5).

Our $P_{m}$ value agrees very well with $P_{m}=43\;µm/s$ that was obtained for solvent depleted planar bilayers [42]. But it tenfold exceeds the one reported by an imaging study of acid uptake into cholesterol containing giant unilamellar vesicles (GUVs) [43]. Conceivably, the tenfold difference has to be attributed to peculiarities of the imaging study. Differences in the membrane compositions are likely to make a minor contribution. We came to that conclusion because $P_{m}$ to acetic acid also differed by an order of magnitude between the imaging study [43] and a previous scanning electrochemical microscopy study [9], although cholesterol was present in both studies.

The neglect of buffers in the imaging study [43] may provide a clue. In contrast to the theoretical analysis of that study, we observed slower kinetics for higher ratios of buffer concentration to ${\left[AH\right]}_{out}$ in our experiment (Figure 4). We have previously observed a similar dependence of the acid/base flux on buffer concentration in our scanning electrochemical microscopy studies [9,10].

**Figure 6:**
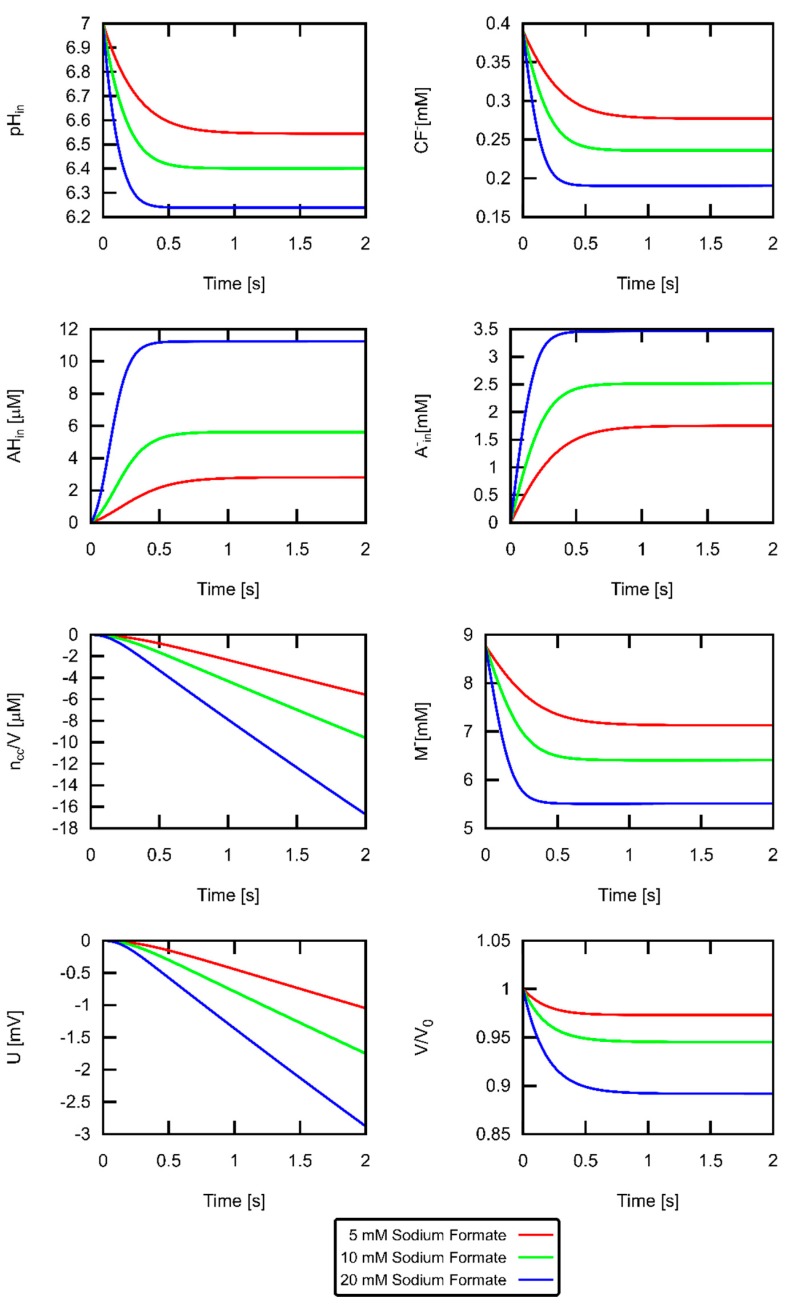
The effect of external weak acid concentration on acid uptake, vesicle volume and membrane voltage. Quantities ($pH_{in}$, concentration of unprotonated dye $\left[CF^{-}\right]$, relative vesicle volume $V/V_{0}$, concentration of protonated and deprotonated acid ${\left[AH\right]}_{in}$, ${\left[A^{-}\right]}_{in}$, net concentration of protons that have permeated $n_{cc}/V$, concentration of unprotonated buffer $\left[M^{-}\right]$, and the transmembrane potential U) extracted from the fit of $I_{theor}$ to $I_{exp}$ in Figure 4 (middle panel, 10 mM MES). The sodium formate gradient is denoted in the key.

To illustrate the importance of buffer effects, we plotted the large changes of intravesicular pH for different weak acid concentrations (Figure 6). For the highest acid concentration (conditions of middle panel of Figure 4), the pH drop halved $\left[M^{-}\right]$. The fluorescence signal is proportional to $\left[CF^{-}\right]$ that also changes with the same time constant. It is important to note that the chemical reactions of $CF^{-}$, $M^{-}$, and $A^{-}$ are not rate limiting in our experiments—as Figure 5 confirms. They take place much faster than the permeation of formic acid. The 10% decrease in volume occurred with a somewhat slower time constant as the pH drop due to the limited water permeability. The changes of the impermeable ${\left[A^{-}\right]}_{in}$ are much more pronounced than those of the permeable ${\left[AH\right]}_{in}$. Finally, we show the time course of how $n_{cc}$ (here depicted as $n_{cc}/V$) charges the membrane, thereby giving rise to U.

In contrast, protons do not reach their electrochemical equilibrium. They leak too slowly across the membrane to significantly affect $\left[CF^{-}\right]$ (and thus $I_{theor}$) on the experimental timescale. Consequently, U is much smaller than proton’s Nernst potential of 30–60 mV (Figure 6). Even a tenfold higher $P_{H^{+}}$ would result in only an insignificant decrease of $P_{m}$ as revealed by our model calculations. To illustrate this fact, Figures S2 and S3 show the corresponding fitting results for the experiment displayed in the middle panel of Figure 4. The tenfold elevated $P_{H^{+}}$ would decrease the calculated $P_{m}$ from 34.9 µm/s to 34.3 µm/s.

Small deviations between the experimental traces and the calculated traces (Figure 4) may be accounted for by vesicle polydispersity. This view is supported by vesicle volume distribution (Figure 2) that shows the presence of vesicles with diameters >100 nm.

**Figure 7:**
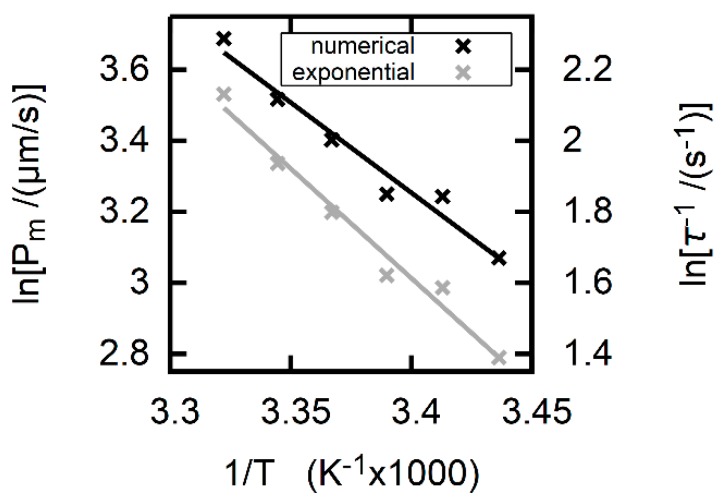
Arrhenius plots for the uptake of formic acid into DOPC vesicles. The temperature increased from 18 °C to 28 °C in steps of 2 °C. The temperature dependence of $P_{m}$ (obtained from fitting $I_{theor}$ to $I_{exp}$) indicates an activation energy $E_{A}=9.8\pm 1.0\;\mathrm{kcal}/\mathrm{mol}$. If calculated from 1/τ (Equation (6)), *E*_A_ amounts to 12.2±0.9 kcal/mol (gray, right axis). The different values indicate that the time constant *τ* of an exponential fit to $I_{theor}$ is not proportional to $P_{m}$, and thus Equation (6) cannot be used to calculate $P_{m}$. The traces and fits are shown in Figure S4. The external concentration of sodium formate was equal to 20 mM. For the remaining experimental conditions please see the legend to the lower panel of Figure 4.

The Arrhenius plot of $P_{m}$ (Figure 7) revealed $E_{A}=9.8\pm 1.0\;\mathrm{kcal}/\mathrm{mol}$ for formic acid permeation through DOPC bilayers. In contrast, the Arrhenius plot of $\tau^{-1}$ yielded $E_{A}=12.2\pm 0.9\;\mathrm{kcal}/\mathrm{mol}$. We obtained $\tau^{-1}$ by fitting the simple mono-exponential function $I\left(t\right)=I_{0}+\Delta I\cdot \exp\left(-t/\tau \right)$ to $I_{exp}$ (Figure S4). The time dependence of $I_{exp}$ and ${\left[AH\right]}_{in}$ (Equation (6)) must be identical if (i) buffer capacity is time invariant (as assumed for the derivation of Equation (6)) and (ii) the CF fluorescence intensity is proportional to pH.

The different $E_{A}$ values indicate that $P_{m}$ is not proportional to $\tau^{-1}$. This observation is in sharp contrast to Equation (6). We conclude that Equation (6) represents an inadequate approximation to the system of Equations (10)–(16), i.e., 1/τ should not be used for calculations of $P_{m}$.

## 4. Discussion

Our study advances the current methodology of determining $P_{m}$ of weak acids by proposing an analytical model that accounts for the significant impact of buffer molecules on acid influx into large unilamellar vesicles. In contrast to previous models, it allows for intravesicular buffer expenditure. This is a “conditio-sine-qua-non” of extracting $P_{m}$ from the time course of intravesicular pH, because buffer molecules are ubiquitously present, e.g., in terms of (i) fluorescent pH indicators, (ii) the permeating weak acid itself, and (iii) additionally added proton acceptors or donors. Our model also accounts for the effects that both the uptake driven decrease in $pH_{in}$ and variations in ${\left[AH\right]}_{out}$ exert on intravesicular buffer capacity.

Most importantly, the model exposes the often assumed proportionality between 1/τ and $P_{m}$ (Equations (6)) as a misconception. Other processes appear to contribute to 1/τ in addition to membrane permeation. In consequence, the values of *E*_A_ that can be derived from the temperature dependencies of 1/τ and $P_{m}$ differ significantly from each other (Figure 7).

An illustrative example for the ill use of a proportionality between 1/τ and $P_{m}$ is provided by investigations of CO_2_ membrane permeability. The corresponding attempts to determine CO_2_ membrane permeability by stopped-flow experiments [7,44] resulted in severe underestimations, i.e., in *P*_m_ values that were orders of magnitude smaller than those determined in experiments under steady state conditions on planar lipid bilayers [45,46,47]. Neither cholesterol-free nor cholesterol-containing bilayers provide a barrier to CO_2_ under the conditions that were chosen for the stopped flow experiments. Calculations to the contrary were flawed by the assumption of a time invariant buffer capacity [44]. Accordingly, a non-realistic delay in CO_2_ uptake kinetics beyond the dead time of the device was predicted. However, in the actual experiment, the buffer was rapidly exhausted. As illustrated by Figure 4, a decreased buffer capacity acts to accelerate uptake kinetics. Accordingly, pH must have dropped much faster than anticipated by the CO_2_ uptake calculations. Our analysis is supported by the observation that cholesterol decreases the permeability to other small molecules like O_2_ [48] and H_2_O [35] less than tenfold. Thus, the thousand-fold decrease in CO_2_ permeability as derived from the stopped flow experiments is unreasonable, unless cholesterol were to act specifically on CO_2_ permeation.

Figure 6 reports that *V* reaches steady state slower than ${\left[AH\right]}_{in}$ does. That is, the osmotic response would not provide a proper read-out for acid uptake kinetics in our experiments. Even if *P*_f_ was enhanced (e.g., by aquaporin reconstitution) to render osmosis non-rate limiting, *τ* was to remain the sole readout parameter. As outlined above, *τ* (Equation (6)) cannot be used to calculate $P_{m}$, because buffer effects on acid flux kinetics are not properly taken into account. Thus, $P_{m}$ values deduced from osmotic measurements [12,49] may not be accurate. Our analysis does not question $P_{m}$ estimates for solutes that do not undergo chemical reactions (e.g., urea [50])

Deprotonation rates of buffer molecules and fluorophores may represent another caveat of vesicular uptake studies. That is, in case of fast permeating acids, the accompanying chemical reactions cannot always be regarded as instantaneous. They may limit the kinetics for substances with neutral or basic pK values, because proton release takes tens or hundreds of milliseconds [18]. In consequence, uptake studies of drugs like propranolol or verapamil (with pK values of 9.5 and 8.9, respectively) [51] may not reveal $P_{m}$, because deprotonation occurs much slower than membrane transport [8]. Our theoretical model explicitly accounts for the reaction rates. That is, the impact of $P_{m}$ on the acidification kinetics may easily be verified (Figure 5).

## 5. Conclusions

Kinetics and extent of the fluorescence intensity response of dye-loaded vesicles to acid gradients depend on luminal buffer concentration (Figure 6). Since fast uptake kinetics requires fast acquisition, filtering and averaging of noisy fluorescent signals is of limited value. In consequence, comparatively large pH changes are induced to detect the signal behind the fluorescence noise. In turn, buffer exhaustion must be taken into account. In addition, calculation of *P*_m_ must generally be performed with respect to (i) the protonation and deprotonation rates of the substance of interest, (ii) proton leak, (iii) osmotic volume flow, and (iv) water self-dissociation.

The formalism presented in this paper is applicable to the permeation of weak acids and beyond. It can easily be expanded to the permeation of other solutes: (α) weak bases and (β) solutes that do not undergo chemical reactions but induce measureable volume changes instead.

## Figures and Tables

**Table 1:** Values of parameters used for computation.

Parameter	Symbol	Value	Unit	Reference
Acid dissociation constant formic acid	$pK_{AH}$	3.75	1	[29]
Temperature shift of $pK_{AH}$	$dpK_{AH}$	0.001	$K^{-1}$	[30]
Deprotonation rate formic acid	$k_{AH}^{+}$	$5.6\cdot 10^{6}$	$s^{-1}$	After [18]
Acid dissociation constant carboxyfluorescein	$pK_{CF}$	6.45	1	[19], Figure S1
Temperature shift of $pK_{CF}$	$dpK_{CF}$	−0.005	$K^{-1}$	Figure S1
Deprotonation rate carboxyfluorescein	$k_{CF}^{+}$	$7.1\cdot 10^{3}$	$s^{-1}$	[31]
Acid dissociation constant MES	$pK_{M}$	6.15		[32]
Temperature shift of $pK_{M}$	$dpK_{M}$	−0.011	$K^{-1}$	[32]
Deprotonation rate MES	$k_{M}^{+}$	$2.2\cdot 10^{4}$	$s^{-1}$	After [18]
Water dissociation rate	$k_{w}^{+}$	$2.5\cdot 10^{-5}$	$s^{-1}$	[33]
Temperature shift of water dissociation constant	$dpK_{W}$	−0.033	$K^{-1}$	Linear approximation in relevant temperature range [34]
DOPC water permeability	$P_{f}$	16	$µm\cdot s^{-1}$	[35]
Specific membrane capacity	c	1	$µF/{\mathrm{cm}}^{2}$	[36]
Membrane proton permeability	$P_{H^{+}}$	$3.5\cdot 10^{-5}$	cm/s	This study

MES: 2-(*N*-morpholino)ethanesulfonic acid, DOPC: 1,2-dioleoyl-*sn*-glycero-3-phosphocholine.

## Supplementary Materials

The following are available online at http://www.mdpi.com/2218-273X/9/2/63/s1. Figure S1: pH titration of carboxyfluorescein (CF) at 22 °C and 4 °C; Figure S2: Evaluation of acid influx with varying proton permeability; Figure S3: Quantities underlying the evaluation of Figure S2. Figure S4: Temperature dependent acid influx traces;
