# Supplementary material for: The Effect of Buffers on Weak Acid Uptake by Vesicles
Source: Biomolecules. 2019 Feb 13;9(2):63. doi: 10.3390/biom9020063 (PMC6406578; doi:10.3390/biom9020063)
Supplement: Supplementary file 1 [file biomolecules-09-00063-s001.pdf]

biomolecules

Supplementary Materials

The effect of buffers on weak acid uptake by vesicles

Christof Hannesschlaeger, Thomas Barta, Hana Pechova and Peter Pohl *

Institute of Biophysics, Johannes Kepler University Linz, Gruberstr. 40, 4020 Linz, Austria; Christof.Hannesschlaeger@jku.at (C.H.); Thomas.Barta@jku.at (T.B.); hanpechova@gmail.com (H.P.)

* Correspondence: Peter.Pohl@jku.at; Tel. +43-732-2468-7562

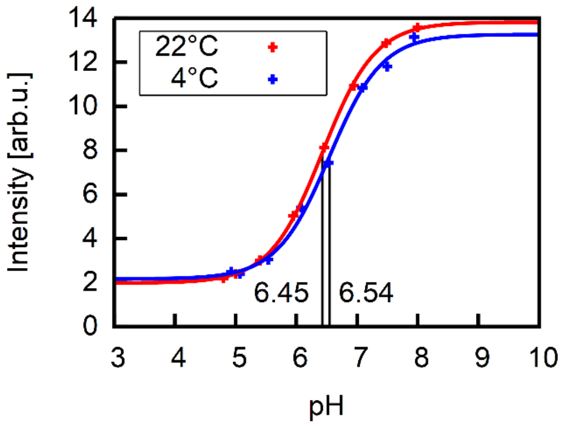

Figure S1. pH titration of carboxyfluorescein (CF) at 22°C and 4°C. 1 μM CF in a 95 mM KCl solution buffered with 5 mM beta alanine, 5mM MES, 5 mM TRIS and 5 mM CAPS. The samples are titrated by HCl addition. The added volumes produce negligible dilution. Emission spectra (slit-width 2.5 nm) are recorded on a fluorescence spectrophotometer with temperature control (HITACHI F2700; Tokyo, Japan). The excitation wavelength is equal to 480 nm (slit-width 2.5 nm). Integration of the spectra from 515 nm to 650 nm mimics the detection of the stopped-flow device that is equipped with a 515 nm longpass filter. The intensities are plotted against the respective pH for 22°C (red) and 4°C (blue). The solid lines represent fits of a function of the form $(pH) = I_0 + \Delta I/(1 + 10^{pK_{CF}-pH})$ to the data. I_0 and ΔI are device dependent with pK_{CF} being equal to 6.45 (22°C) or 6.54 (4°C). That is, the temperature induced shift of dpK_{CF}.amounts to $-0.005\ \mathrm{K}^{-1}$.

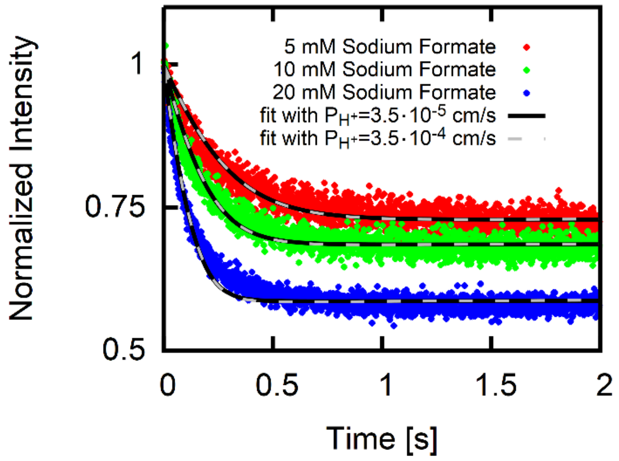

Figure S2. Proton permeability P_{H^+} is too small to significantly affect intravesicular acidification. Two fits of I_{theor} (black and gay lines) to every I_{exp} (colored lines) in the presence of 10 mM MES (Figure 4, middle panel) are displayed. The black line is obtained by assuming $P_{H^+} = 3.5 \cdot 10^{-5}$ cm/s . The gray line dashed line assumes $P_{H^+} = 3.5 \cdot 10^{-4}$ cm/s.

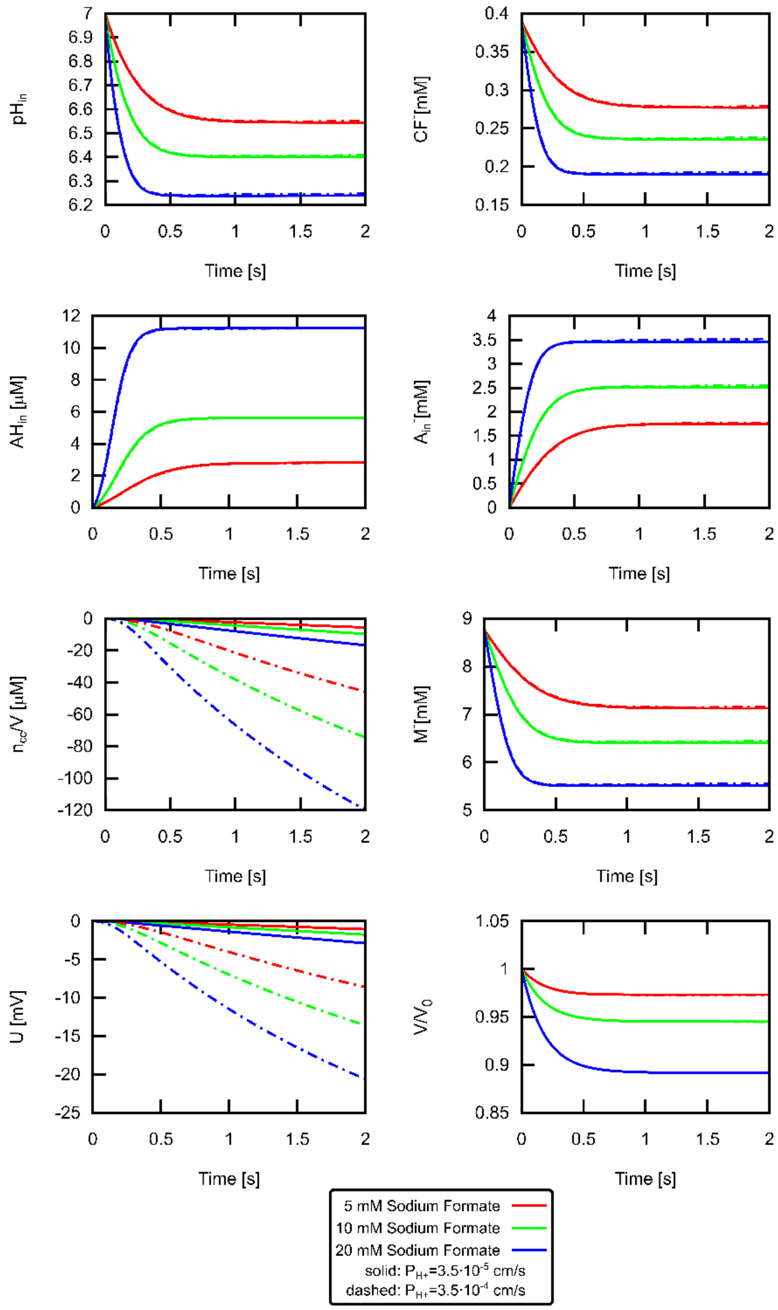

Figure S3. Parameter sensitivity to variation of P_{H^+}. Results of the numerical calculations performed for Figure S2 are shown: solid lines for $P_{H^+} = 3.5 \cdot 10^{-5}$ cm/s 3; dashed lines for $P_{H^+} = 3.5 \cdot 10^{-4}$ cm/s.

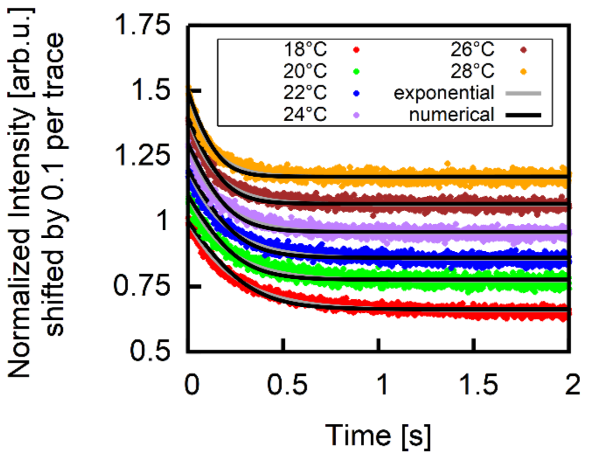

Figure S4. Temperature dependence of acid uptake into DOPC vesicles. Formic acid influx induced intravesicular acidification depends on temperature as indicated by CF fluorescence intensity. The traces have been used for calculation of Figure 7 in the main text. For experimental conditions see lower panel of **Error! Reference source not found.**. Maintaining an invariant 20 mM sodium formate gradient, the temperature was incremented in 2°C steps from 18°C to 28°C. The normalized traces are shifted by 0.1 arbitrary units for displaying purposes. I_{theor} (black lines) is fitted to I_{exp} (colored lines) to obtain P_m. In addition, the exponential function $I(t) = I_0 + \Delta I \cdot \exp(-t/\tau)$ is also fitted to the data (grey line) to obtain the exponential time constant τ. P_m and τ are plotted in Figure 7 of the main text.

© 2019 by the authors. Licensee MDPI, Basel, Switzerland. This article is an open access article distributed under the terms and conditions of the Creative Commons Attribution (CC BY) license (http://creativecommons.org/licenses/by/4.0/).
